# Metabolic Stress Index Including Mitochondrial Biomarker for Noninvasive Diagnosis of Hepatic Steatosis

**DOI:** 10.3389/fendo.2022.896334

**Published:** 2022-05-19

**Authors:** Jae Seung Chang, Jhii-Hyun Ahn, Seong Hee Kang, Sang-Baek Koh, Jang-Young Kim, Soon Koo Baik, Ji Hye Huh, Samuel S. Lee, Moon Young Kim, Kyu-Sang Park

**Affiliations:** ^1^Mitohormesis Research Center, Yonsei University Wonju College of Medicine, Wonju, South Korea; ^2^Department of Physiology, Yonsei University Wonju College of Medicine, Wonju, South Korea; ^3^Department of Radiology, Yonsei University Wonju College of Medicine, Wonju, South Korea; ^4^Department of Internal Medicine, Yonsei University Wonju College of Medicine, Wonju, South Korea; ^5^Regeneration Medicine Research Center, Yonsei University Wonju College of Medicine, Wonju, South Korea; ^6^Department of Preventive Medicine, Yonsei University Wonju College of Medicine, Wonju, South Korea; ^7^Department of Internal Medicine, Hallym University Sacred Heart Hospital, Anyang, South Korea; ^8^Liver Unit, University of Calgary Cumming School of Medicine, Calgary, AB, Canada

**Keywords:** mitochondria, non-alcoholic fatty liver disease, FGF21, FGF19, adiponectin-to-leptin ratio, central obesity, MRI-PDFF, biopsy-proven fatty liver

## Abstract

**Background:**

Mitochondrial dysfunction with oxidative stress contributes to nonalcoholic fatty liver disease (NAFLD) progression. We investigated the steatosis predictive efficacy of a novel non-invasive diagnostic panel using metabolic stress biomarkers.

**Methods:**

Altogether, 343 subjects who underwent magnetic resonance imaging-based liver examinations from a population-based general cohort, and 41 patients enrolled in a biopsy-evaluated NAFLD cohort, participated in the development and validation groups, respectively. Serologic stress biomarkers were quantitated by enzyme-linked immunosorbent assay.

**Results:**

Multivariate regression showed that waist-to-hip ratio, fibroblast growth factor (FGF) 21, FGF19, adiponectin-to-leptin ratio, insulin, albumin, triglyceride, total-cholesterol, and alanine-aminotransferase were independent predictors of steatosis (rank-ordered by Wald). The area under receiver-operator characteristics curve [AUROC (95%CI)] of the metabolic stress index for steatosis (MSI-S) was 0.886 (0.85−0.92) and 0.825 (0.69−0.96) in development and validation groups, respectively. MSI-S had higher diagnostic accuracy (78.1%−81.1%) than other steatosis indices. MSI-S notably differentiated steatosis severities, while other indices showed less discrimination.

**Conclusion:**

MSI-S, as a novel non-invasive index, based on mitochondrial stress biomarker FGF21 effectively predicted steatosis. Furthermore, MSI-S may increase the population that could be excluded from further evaluation, reducing unnecessary invasive investigations more effectively than other indices.

## Introduction

Nonalcoholic fatty liver disease (NAFLD) is the most prevalent chronic liver disease, progressing from simple steatosis to NASH, fibrosis, cirrhosis, and hepatocellular carcinoma. Simple hepatic steatosis has a benign nature, whereas NASH is more likely to progress to cirrhosis and cancer (1). Approximately 10−29% of patients with NASH develop cirrhosis within 10 years; of these, 4−27% develop hepatocellular carcinoma (2). Because of the high prevalence and serious progression, reliable diagnostic and prognostic strategies for NAFLD represent an important unmet need. To date, however, there is no simple bloodwork panel or scoring system for the non-invasive evaluation of NAFLD disease severity and therapeutic plans (3).

The pathogenesis of NAFLD includes oxidative and metabolic stresses associated with obesity and insulin resistance; thus, NAFLD is often considered the liver manifestation of metabolic syndrome (4). A diet high in carbohydrates and fat with physical inactivity leads to free fatty acid and triglyceride accumulation in the liver, causing reactive oxygen species (ROS) production in the cytosol and mitochondria (5, 6). Subsequently, this oxidative stress inflicts prolonged mitochondrial and endoplasmic reticulum (ER) stress, which leads to further excessive ROS generation from the mitochondria and ER (7). The ‘vicious cycle’ between oxidative stress and organellar dysfunction leads to adverse effects, including hepatic inflammation and cytotoxicity (8).

In reaction to mitochondrial and ER stresses, cells show adaptive and protective responses including increased mitochondrial biogenesis, improved bioenergetic status, and upregulated antioxidant defence and quality control systems (9). These recovery actions are partly mediated by the integrated stress response (ISR) that induces fibroblast growth factor-21 (FGF21) and growth differentiation factor-15 (GDF15) *via* upregulation of activating transcription factor 4 (10, 11). FGF21 and GDF15, as humoral factors of ISR, are secreted from the liver and other tissues and play a protective role against mitochondrial injury and metabolic exacerbation (12, 13). Therefore, ISR contributes to metabolic resilience and flexibilities against mitochondrial stress, which could abate or delay the onset of NAFLD (10, 14).

Intriguingly, it has been demonstrated that hepatic mitochondrial respiration is activated in patients with obesity-related fatty liver who have elevated serum FGF21 concentrations. This mitochondrial adaptation is explained as hepatic mitochondrial flexibility to simple steatosis (15). However, patients with obesity with progression to NASH fail to adapt to pathologic stresses leading to hepatic mitochondrial dysfunction with higher proton leak, oxidative stress, and inflammation, even under further elevated FGF21 levels (15). Despite the recovery efforts against mitochondrial stress, in uncompensated pathologic conditions, sustained stress continues to stimulate the induction and maintain higher serum levels of these factors.

Serum biomarker analyses have revealed elevated FGF21 levels in patients with metabolic disease related to obesity and insulin resistance (16, 17). Particularly, patients with NAFLD, including fatty liver and steatohepatitis, have significantly higher serum FGF21 values than control subjects, which could prove useful for the non-invasive prediction of steatosis and fibrosis status in NAFLD (18). However, the serum FGF21 concentrations are highly variable between individuals, which may hamper the usefulness of this single indicator (19, 20). Therefore, in our study, we obtained serum values of a panel of biomarkers, including FGF21, for multiplexed and contextual evaluation of NAFLD. We demonstrated that inclusion of this mitochondrial stress biomarker significantly improves the usefulness and effectiveness of algorithms for estimating steatosis.

## Methods

### Study Participants

**Figure 1:**
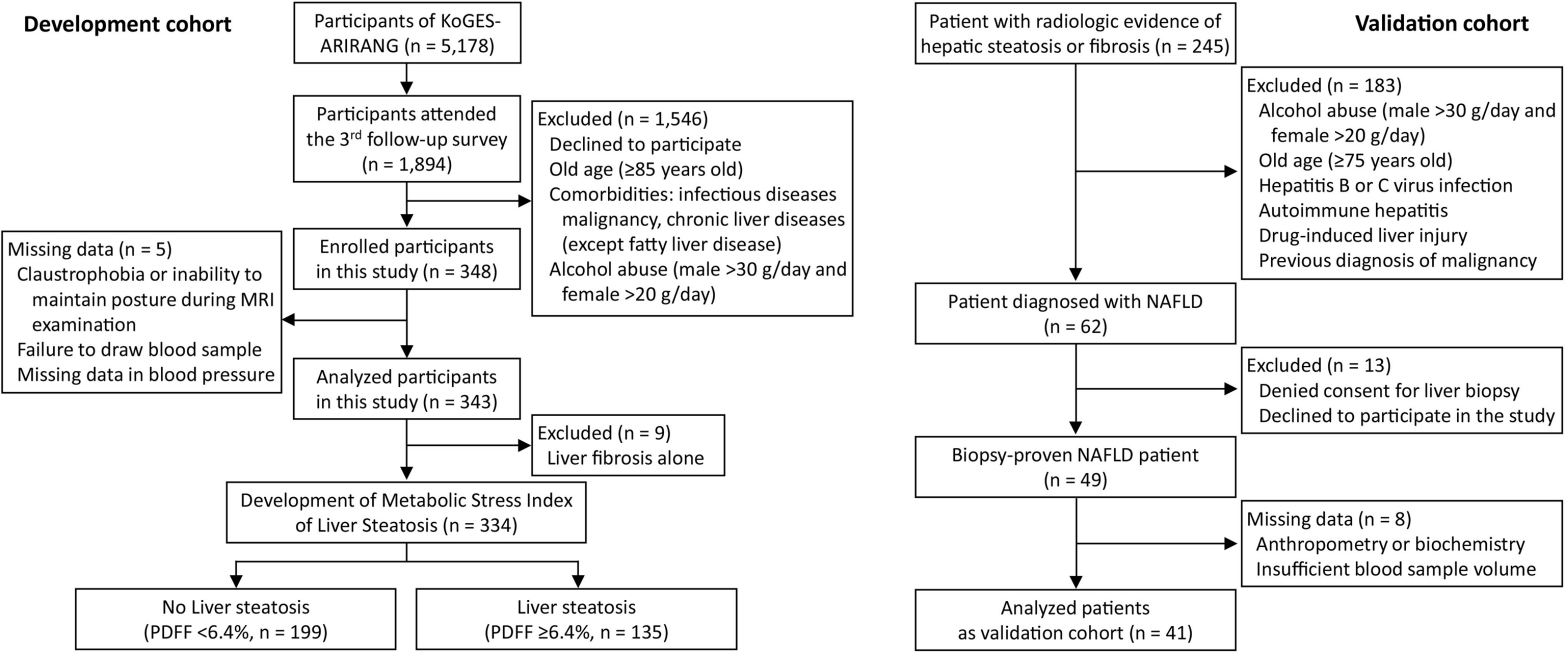
Flow chart of participant recruitment and analyzed subgroups in this study. KoGES-ARIRANG, Korean Genome and Epidemiology Study on the Atherosclerosis Risk of Rural Areas in the Korean General Population; MRI-PDFF, magnetic resonance imaging-proton density fat fraction.

The present study comprised 343 volunteers (124 men and 219 women) recruited from a population-based general cohort, KoGES-ARIRANG (the Korean Genome and Epidemiology Study on Atherosclerosis Risk of Rural Areas in the Korean General Population) which comprised the development cohort, and 41 patients (16 men and 25 women) of a biopsy-evaluated NAFLD cohort for the validation cohort (21). Study population recruitment and selection procedures are detailed in **Figure 1** and **Supplementary Methods**. The study was conducted in accordance with the ethical guidelines of the 1975 Helsinki Declaration, and was approved by the institutional review board of Wonju Severance Christian Hospital (IRB No. CR317131 and CR318003). All study participants were informed about the rationale and possible risks of the study and provided written informed consent before participation.

### Evaluation of Hepatic Steatosis

Quantitative assessment of MRI-based proton density fat fraction (MRI-PDFF) was performed in the development cohort. Hepatic steatosis was graded according to the following criteria: grade 0, PDFF <6.4%; grade 1, 6.4%≤ PDFF<16.3%; grade 2, 16.3%≤ PDFF <21.7%; grade 3, PDFF ≥21.7% (22, 23). Histological examinations of liver biopsy specimens were carried out in the validation cohort and their features were classified according to criteria outlined by Kleiner et al. (24). Steatosis was graded using 4 grades (S0, <5%; S1, 5%−33%; S2, 33%−66%; S3, >66%) based on the percentage of fat-containing hepatocytes. Further detailed diagnostic processes are provided in **Supplementary Methods**.

### Clinical and Laboratory Assessments

Anthropometric measurements such as weight, height, and waist and hip circumference were taken, and then the body mass index (BMI), waist-to-height ratio (WHtR), and waist-to-hip ratio (WHR) were calculated. Routine biochemical tests, including parameters of liver test, were performed using automated clinical chemistry analysers. Serum concentrations of 10 metabolic stress-related biomarkers containing FGF21 and GDF15 were quantified by using commercially available ELISA kits according to the manufacturer’s instructions. Ultrasonography was conducted in all participants to determine fatty liver severity. The details of clinical and laboratory assessment and the criteria for metabolic diseases are provided in the **Supplementary Methods**. We also calculated several predictive scores derived from clinical and laboratory indices to compare their diagnostic performance in liver steatosis. The details of clinico-laboratory assessments, the criteria of metabolic diseases, and the scoring formulae are provided in the **Supplementary Methods**.

### Statistical Analysis

Continuous data are presented as medians with interquartile ranges (IQR) and the categorical data are presented as frequencies with proportions. All variables collected in the development cohort were included in a multivariate forward stepwise logistic regression analysis to identify variables independently associated with presence or absence of NAFLD. Non-parametric data were used as independent variables after natural logarithmic transformation. The contribution strength of each variable to the multivariate model was evaluated by the Wald chi-square value (Wald χ^2^), which was calculated by squaring the ratio of the regression coefficient divided by its standard error. The diagnostic powers of prediction models were evaluated by area under the receiver operator characteristic curve (AUROC) analyses with assessments of likelihood ratios, predictive values, and diagnostic accuracy. Several cut-off values were calculated for the diagnosis of steatosis: the one that corresponded to the highest Youden index, which maximizes sensitivity and specificity, and the others that corresponded to ≥90% sensitivity (low threshold for ruling-out) and ≥90% specificity (upper threshold for ruling-in). All statistical tests were 2-tailed and *p* values <0.05 were considered significant. Data were analyzed using IBM SPSS Statistics for Windows, version 25.0 (IBM Corp., Armonk, NY, USA). For further details regarding the statistical methods used, please refer to the **Supplementary Methods**.

## Results

### Baseline Characteristics of the Study Participants

The median (IQR) age and BMI in the development and validation cohorts were 66 years (61−72) and 25.0 kg/m^2^ (23.2−27.5), and 47 years (33−59) and 27.7 kg/m^2^ (25.9−31.3), respectively. Of the development cohort, 39.4% of participants had NAFLD (MRI-PDFF ≥6.4%). In the validation cohort, 61.0% of subjects were diagnosed with biopsy-proven moderate-to-severe steatosis. The clinical and laboratory characteristics of the subjects are described in **Supplementary Tables S1, S2**.

### Development of Metabolic Stress Index for Liver Steatosis

Independent predictive variables derived from logistic regression analyses with NAFLD as a dependent variable are described in **Table 1**. In the univariate logistic analyses, mitochondrial stress biomarkers correlated with liver fat content with a high significance coefficient (Wald χ^2^; 42.2 for FGF21, *p <*0.001), in addition to central obesity (Wald χ^2^; 43.5 for WHR, *p <*0.001). Serum FGF19 and adiponectin-to-leptin ratio (A/L) were negatively correlated with steatosis, consistent with previous findings (25, 26). In the multivariate logistic analysis, WHR and natural logarithms of FGF21, FGF19, A/L, insulin, albumin, total triglycerides (TG), total cholesterol (TC), and aminotransferase (ALT) were selected as significant independent predictors (rank-ordered by Wald χ^2^) and used to develop a metabolic stress index for steatosis (MSI-S): e^x^/(1 + e^x^) · 100.


$$\begin{array}{l} \text{x}=14.079\cdot \text{WHR}+0.888\cdot \text{In}(\text{FGF}21,\text{pg}/\text{mL})-0.579\cdot \text{In}(\mathrm{FGF}19,\text{pg}/\text{mL})-0.469\cdot \text{In}(\text{A}/\text{L},10^{3})\;+\\ 0.652\cdot \text{In}(\text{insulin},\text{mU}/\text{L})+8.06\cdot \text{In}(\text{albumin},\text{g}/\text{dL})+0.08\cdot \text{In}(\text{TG},\text{mg}/\text{dL})+1.878\cdot \text{In}(\text{TC},\text{mg}/\text{dL})\;+\\ 0.833\cdot \text{In}(\text{ALT},\text{IU}/\text{L})-45.426 \end{array}$$

**Figure 2:**
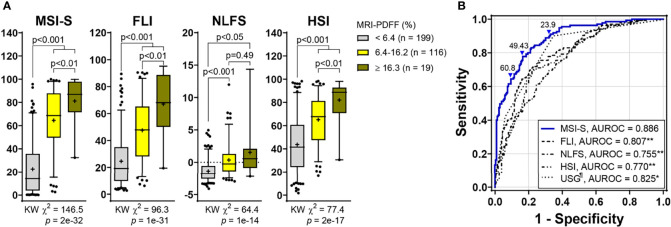
Predictive ability of MSI-S for liver fat content compared with other steatosis indices. **(A)** Non-invasive prediction scores according to fatty liver grades (Kruskal-Wallis [KW] test with *post hoc* Dunnett’s T3 test). Data are presented as box and whisker Tukey plots with medians and interquartile ranges (+, mean; •, outliers). **(B)** ROC curves of non-invasive scores for predicting hepatic steatosis. The optimal cutoff value determined using the Youden Index was 49.43. The cutoffs to achieve a ≥90% sensitivity and a ≥90% specificity were 23.9 (low threshold for ruling-out) and 60.8 (high threshold for ruling-in), respectively. *p < 0.002, **p < 0.001 vs. MSI-S; ^¶^missing data (n = 2) (DeLong’s tests). MSI-S, metabolic stress index of liver steatosis; FLI, fatty liver index; NLFS, NAFLD liver fat score; HSI, hepatic steatosis index; USG, ultrasonography; MRI-PDFF, magnetic resonance imaging-proton density fat fraction; AUROC, area under the ROC (receiver operating characteristic) curve.

The Hosmer-Lemeshow statistic was not significant (χ^2^ = 4.24, *p* = 0.835) and the Nagelkerke *R*^2^ was 0.547, indicating good fitness of the MSI-S model. The AUROC of the predicted probability value for the MSI-S was 0.886 (95% CI 0.85−0.92), which shows excellent diagnostic performance compared with previously suggested indices for fatty liver, including the fatty liver index (FLI) [AUROC (95% CI); 0.807 (0.76−0.85)], NAFLD liver fat score (NLFS) [0.755 (0.70−0.81)], and hepatic steatosis index (HSI) (0.770 [0.72−0.82]) (**Figure 2**). Compared with ultrasonography [0.825 (0.78−0.87)], the MSI-S also showed a higher AUROC [0.884, (0.85−0.92)] in participants who had undergone ultrasonography. At the optimal cut-off value of 49.43, the MSI-S could rule out NAFLD with a sensitivity of 78% (95% CI 70−83) and a negative likelihood ratio of 0.27 (95% CI 0.19−0.37), and detect NAFLD with a specificity of 83% (95% CI 78−88) and a positive likelihood ratio of 4.6 (95% CI 3.4−6.5). Consequently, the MSI-S has a higher diagnostic accuracy (81.1%) than other steatosis indices (**Table 2**).

**Table 1:** Univariate and multivariate (stepwise forward) logistic regression analyses for the prediction of hepatic steatosis.

	Univariate				Multivariate			
Variable	Coefficient (95% CI)	S.E.	Wald	*P*–value	Coefficient (95% CI)	S.E.	Wald	*P*–value
Body mass index (kg/m^2^)	0.27 (0.19 to 0.35)	0.1	41.1	<0.001				
Ln [waist (cm)]	8.77 (6.15 to 11.39)	1.3	43	<0.001				
Waist-to-height ratio	14.9 (10.1 to 19.7)	2.5	36.7	<0.001				
Waist-to-hip ratio	16.2 (11.4 to 21.0)	2.5	43.5	<0.001	14.079 (7.731 to 20.427)	3.24	18.89	<0.001
Ln [triglyceride (mg/dL)]	1.51 (1.02 to 2.0)	0.3	36.8	<0.001	0.808 (0.171 to 1.445)	0.33	6.18	0.013
Ln [total cholesterol (mg/dL)]	1.15 (0.04 to 2.27)	0.6	4.1	0.043	1.878 (0.32 to 3.436)	0.8	5.58	0.018
Ln [high–density lipoprotein (mg/dL)]	–1.56 (–2.42 to –0.71)	0.4	12.8	<0.001				
Ln [fasting glucose (mg/dL)]	1.47 (0.26 to 2.68)	0.6	5.67	0.017				
Ln [fasting insulin (mU/L)]	1.14 (0.8 to 1.49)	0.2	42	<0.001	0.652 (0.166 to 1.138)	0.25	6.9	0.009
Ln (HOMA–IR)	0.96 (0.66 to 1.26)	0.2	39.2	<0.001				
Ln [AST (IU/L)]	1.27 (0.46 to 2.08)	0.4	9.42	0.002				
Ln [ALT (IU/L)]	1.84 (1.18 to 2.5)	0.3	29.6	<0.001	0.833 (0.018 to 1.648)	0.42	4.01	0.045
Ln [ALT/AST]	2.22 (1.34 to 3.11)	0.5	24.2	<0.001				
Ln [γ-glutamyltransferase (IU/L)]	1.28 (0.86 to 1.71)	0.2	34.4	<0.001				
Ln [albumin (g/dL)]	6.15 (1.46 to 10.8)	2.4	6.59	0.01	8.206 (1.816 to 14.596)	3.26	6.34	0.012
Ln [uric acid (mg/dL)]	1.39 (0.52 to 2.26)	0.4	9.85	0.002				
Ln [protein (g/dL)]	5.68 (1.49 to 9.87)	2.1	7.07	0.008				
Ln [calcium (mg/dL)]	11.5 (5.14 to 17.8)	3.2	12.6	<0.001				
Ln [C–Peptide (ng/mL)]	1.37 (0.93 to 1.82)	0.2	36.5	<0.001				
Ln [GDF15 (pg/mL)]	0.84 (0.34 to 1.34)	0.3	10.8	0.001				
Ln [FGF21 (pg/mL)]	1.27 (0.89 to 1.65)	0.2	42.2	<0.001	0.888 (0.418 to 1.358)	0.24	13.72	<0.001
Ln [FGF19 (pg/mL)]	–0.54 (–0.84 to –0.25)	0.2	13	<0.001	–0.579 (–0.967 to –0.191)	0.2	8.56	0.003
Ln [adiponectin (μg/mL)]	–0.87 (–1.2 to –0.54)	0.2	27.4	<0.001				
Ln [leptin (ng/mL)]	0.69 (0.4 to 0.99)	0.2	21.6	<0.001				
Ln [A/L (10^3^)]	–0.84 (–1.1 to –0.59)	0.1	42.7	<0.001	–0.469 (–0.808 to –0.13)	0.17	7.36	0.007
Ln [RBP4 (μg/mL)]	0.89 (0.1 to 1.69)	0.4	4.87	0.027				
Ln [interleukin 6 (pg/mL)]	0.46 (0.14 to 0.79)	0.2	7.67	0.006				
Constant					–45.426 (–61.192 to –29.66)	8.04	31.89	<0.001

ln, natural logarithm; HOMA-IR, homeostatic model assessment of insulin resistance; AST, aspartate-aminotransferase; ALT, alanine-aminotransferase; γ-GT, γ-glutamyltransferase; ALP, alkaline-phosphatase; GDF15, growth differentiation factor 15; A/L, adiponectin-to-leptin ratio; RBP4, retinol binding protein 4.

**Table 2:** Diagnostic performance of non-invasive prediction scores for hepatic steatosis.

Development cohort	SN	SP	LR+	LR−	PPV	NPV	Accuracy
MSI-S	77.8 (70−85)	83.4 (78−88)	4.6 (3.4−6.5)	0.27 (0.2−0.4)	76.1 (70−82)	84.7 (80−88)	81.1 (77−85)
FLI	70.4 (62−78)	78.9 (73−84)	3.3 (2.5−4.5)	0.38 (0.3−0.5)	69.3 (63−75)	79.7 (75−84)	75.5 (71−80)
NLFS	86.7 (80−92)	51.3 (44−58)	1.8 (1.5−2.1)	0.26 (0.2−0.4)	54.7 (51−59)	85.0 (78−90)	65.6 (60−71)
HSI	68.9 (60−77)	77.9 (72−84)	3.1 (2.3−4.1)	0.40 (0.3−0.5)	67.9 (62−74)	78.7 (74−83)	74.3 (69−79)
Validation cohort	SN	SP	LR+	LR−	PPV	NPV	Accuracy
MSI-S	96.0 (80–99)	50.0 (25–75)	1.9 (1.2–3.2)	0.08 (0.01–0.6)	75.0 (65–83)	88.9 (52–98)	78.1 (62–89)
FLI	84.0 (64–96)	31.3 (11–59)	1.2 (0.8–1.8)	0.51 (0.2–1.6)	63.3 (54–72)	58.0 (30–82)	62.1 (46–77)
NLFS	100 (86–100)	18.8 (4–46)	1.2 (1.0–1.6)	–	63.5 (58–69)	100	66.3 (50–80)
HSI	92.0 (74–99)	18.8 (4–46)	1.1 (0.9–2.3)	0.43 (0.1–2.3)	61.5 (55–68)	62.4 (24–90)	61.6 (45–76)

Data are presented as percentages (95% CI). MSI-S, metabolic stress index for liver steatosis; FLI, fatty liver index; NLFS, NAFLD liver fat score; HSI, hepatic steatosis index; SN, sensitivity; SP, specificity; LR+, positive likelihood ratio; LR−, negative likelihood ratio; PPV, positive predictive value; NPV, negative predictive value.

Comparing analyses according to the steatosis grade, the Kruskal-Wallis chi-square value (KW χ^2^ = 146.5; *p* = 2e-32) for the MSI-S was higher than those for other indices. MSI-S values for severe steatosis were significantly higher than those for the mild grade [median (95% CI); 68.6 (59.6−69.4) for grade 1 vs. 86.9 (71.6−90.7) for grade 2; *p* = 0.009] (**Figure 2**).

### Validation of Metabolic Stress Index for Liver Steatosis

**Figure 3:**
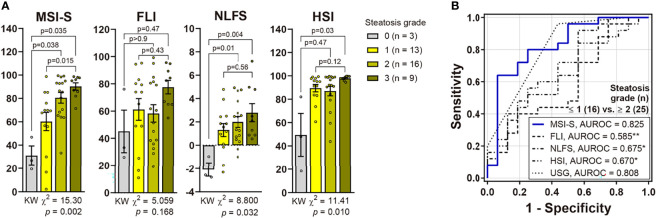
Validation of MSI-S in patients with biopsy-proven NAFLD. **(A)** Non-invasive prediction scores according to histological steatosis grades (Kruskal-Wallis [KW] test with *post hoc* Dunnett’s T3 test). Bars and circles represent the mean with standard error of the mean and individual values, respectively. **(B)** ROC curves of non-invasive scores for predicting moderate-to-severe steatosis. *p < 0.05, **p < 0.01 vs. MSI-S (DeLong’s tests).

Of the validation subjects, median (IQR) values of MSI-S were 26.8 (19.5−46.8) in no steatosis, 69.4 (42.9−80.5) in mild steatosis and 87.0 (71.0−96.9) in moderate-to-severe steatosis. The AUROC of MSI-S for detecting biopsy-proven moderate steatosis (S0−S1 vs. ≥ S2) was 0.825 (95% CI, 0.688−0.962), which indicates superior diagnostic performance compared with other liver steatosis indices (**Figure 3**). By applying the optimal cut-off point derived from the development cohort, the MSI-S had consistently higher diagnostic accuracy (78.1% [95% CI 62.4−89.4]) than other steatosis indices those which all showed an accuracy of less than 70% (**Table 2**). Of predictive steatosis indices, MSI-S values for biopsy-proven severe steatosis were solely significantly higher than those for the mild grade [median (95% CI); 95.1 (83.9−97.8) vs. 69.4 (42.9−80.5); *p* = 0.009)] (**Figure 3**). By comparison, the values of other steatosis indices were not significantly different between the grades of severe and mild steatosis.

### Clinical Applicability of Metabolic Stress Index for Liver Steatosis

**Figure 4:**
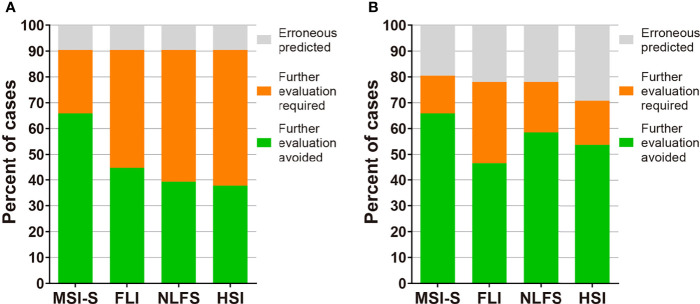
The potential clinical utility model of MSI-S. **(A)** Clinical utility of MSI-S and other indices predicting hepatic steatosis with sensitivity and specificity of 90% in the development cohort. **(B)** Clinical utility of the indices in the validation cohort by applying thresholds derived from the development cohort. Green, true positive and true negative; orange, indeterminate; grey, false positive and false negative.

To improve the clinical applicability of the predictive index, we performed further analyses assuming that a liver biopsy would not be necessary in those who had a true positive or true negative. Cut-off values of MSI-S with a sensitivity and specificity of ≥90% were 23.9 and 60.8, respectively; thus, 65.9% of cases in both the development and validation groups would have avoided a liver biopsy or further investigations. Therefore, MSI-S is more effective to reduce unnecessary invasive examinations than other currently available indices (**Figure 4** and **Supplementary Table S4**).

## Discussion

Here, we developed a novel index for the non-invasive evaluation of steatosis using metabolic stress biomarkers along with NAFLD-related parameters. Compared with previously suggested indices, the MSI-S showed superior predictiveness for hepatic steatosis. Of note, FGF21, as a mitochondrial stress indicator, has high association with fat content and contributed significantly to the high predictive power of this index. These results emphasize the importance of mitochondrial stress in the progression of NAFLD into serious steatosis.

In the present study, quantitative analysis of liver fat content was performed using MRI-PDFF, which is considered the most accurate non-invasive diagnostic modality for steatosis (3). MRI-PDFF represents the proportion of the mobile proton density attributable to fat composition, providing objective evaluation of the amount of hepatic fat. Even though MRI-PDFF is reasonably precise and is used as reference standards, its high cost, time-consuming processes, and unavailability in many global regions preclude widespread applicability in the general population, especially for screening large cohorts.

Major biomarkers in our index are FGF21, FGF19 and A/L, which have not been used in previous biomarker panels for the prediction of fatty liver. FGF21 is upregulated by physiologic and pathophysiologic stresses. In a pathologic condition such as metabolic syndrome, FGF21 increase to compensate for oxidative stress, ER stress, and mitochondrial dysfunction; thus, higher serum levels reflect uncompensated metabolic stress (12). In our study, FGF21 correlated with waist circumference, triglycerides, total cholesterol, γ-GT, and the homeostatic model assessment of insulin resistance, which are identified as the main risk factors of NAFLD. FGF21 was one of the strongest independent predictors for the severity of steatosis.

FGF19, mainly expressed in the small intestine, regulates bile acid synthesis and nutrient metabolism. Additionally, FGF19 has proliferative and anti-apoptotic effects on the liver; therefore, low serum FGF19 levels are associated with hepatocyte injury and cell death (25). Decreased circulating FGF19 levels have been reported in patients with obesity and insulin-resistance (27). Consistently, in our study, FGF19 showed negative correlation with steatosis and enhanced the predictiveness of MSI-S, demonstrating the second highest influence on FGF21.

Hypoadiponectinemia and hyperleptinemia are considered common laboratory findings in NAFLD (28, 29). However, the clinical applicability of the ratio between adiponectin and leptin has not been extensively validated. In our univariate analyses, the A/L ratio had a markedly higher Wald score (42.74) than that of adiponectin (27.39) or leptin (21.57). Furthermore, this ratio was able to discriminate between severe and mild steatosis, whereas adiponectin or leptin alone were not (**Supplementary Figure S1**).

Despite significant correlation with steatosis, several factors such as IL-6, were not used in our index (MSI-S). IL-6 is a well-known inflammatory cytokine, mirroring the severity of steatohepatitis (30). In this study, invasive diagnosis of NASH was not performed; thus, we were unable to evaluate the precise inflammatory status of the NAFLD patients. Instead, serum IL-6 levels had significant correlations with liver fat content (**Table 1** and **Supplementary Table S5**), and increased IL-6 reflected the severity of steatosis (**Supplementary Figure S1**). These results suggest the possible contribution of inflammation to the progression of steatosis.

A positive correlation between central obesity and steatosis was detected in this study, compatible with the pathogenic importance of visceral fat accumulation. Systemic inflammation by visceral obesity is thought to accentuate the progression of NAFLD. Prospective long-term studies in patients with simple liver steatosis are required to elucidate the parameters of the NAFL-NASH-cirrhosis progression in this population.

One striking advantage of MSI-S was its effectiveness in reducing unnecessary further investigations including liver biopsy and MR studies. Compared to currently available indices for hepatic steatosis, in MSI-S the populations of patients with ‘indeterminate’ scores were markedly less, as shown in **Figure 4** and **Supplementary Table S4**. We suggest that MSI-S is an efficient tool for the prediction of steatosis in clinical settings where MR equipment or invasive diagnostic methods are inaccessible.

A limitation of our study was that the validation of steatosis for the general cohort was MR image-based evaluation, not invasive liver biopsy. In fact, it is ethically unfeasible to obtain biopsy samples from healthy cohort subjects. However, as described above, MR-based approaches have advantages in estimating the overall conditions of the liver. Furthermore, we also validated the predictability of MSI-S with liver biopsy-evaluated patients, which was superior to other indices. As another limitation, this study did not encompass an assessment of the wide range of severities of liver steatosis, and this is an opportunity for further validation in larger scale clinical studies. This study may have limitations for gender, age, and ethnicity of the cohort; however, the discriminatory powers of MSI-S were not different between men and women (AUROC [95% CI], 0.914 [0.865–0.964] for men vs. 0.860 [0.811–0.909] for women; DeLong test, z-statistic = 1.53; *p* = 0.13). Further researches involving other races and ethnicity are warranted before definitive conclusions can be made pertaining to the clinical application of MSI-S for identifying patients with a high risk of hepatic steatosis.

In conclusion, we suggest that biomarkers based on the pathophysiologic mechanisms of NAFLD could have predictive power in steatosis, inflammation, and fibrosis. Each biomarker may not, by itself, be suitable as an independent predictor, but integrative interpretation with multivariate logistic regression analyses results in a more reliable index, effective for the non-invasive diagnosis of NAFLD progression. Inclusion of mitochondrial stress marker in the algorithm for steatosis markedly enhanced predictive performance, resulting in more accurate and precise index than other existing scoring systems. As the next step, the MSI-S should be validated in an external population and its prognostic utility needs to be confirmed by other prospective cohort studies.

## Data Availability Statement

The raw data supporting the conclusions of this article will be made available by the authors, without undue reservation, to any qualified researcher.

## Ethics Statement

The studies involving human participants were reviewed and approved by Institutional review board of Wonju Severance Christian Hospital (IRB No. CR317131 and CR318003). The patients/participants provided their written informed consent to participate in this study.

## Author Contributions

JSC, MYK and K-SP conceptualized and designed the study; JSC, J-HA, JHH, SHK, S-BK, J-YK and MYK have conducted the study; JSC, J-HA, JHH, SHK, S-BK, J-YK, MYK and K-SP have collected and interpreted data, JSC, MYK, K-SP, SKB and SSL have drafted the manuscript; JSC, MYK, K-SP, SKB and SSL have revised and finalized the manuscript. All authors contributed to the article and approved the submitted version.

## Funding

This research was funded by the Medical Research Center Program (NRF-2017R1A5A2015369) and in part by the Basic Science Research Program (NRF-2018R1C1B6005036) from the Ministry of Science and ICT, Republic of Korea.

## Conflict of Interest

The authors declare that the research was conducted in the absence of any commercial or financial relationships that could be construed as a potential conflict of interest.

## Publisher’s Note

All claims expressed in this article are solely those of the authors and do not necessarily represent those of their affiliated organizations, or those of the publisher, the editors and the reviewers. Any product that may be evaluated in this article, or claim that may be made by its manufacturer, is not guaranteed or endorsed by the publisher.

## Abbreviations

A/L, adiponectin-to-leptin ratio; ALT, alanine aminotransferase; AUROC, area under the receiver operator characteristic curve; ER, endoplasmic reticulum; FLI, fatty liver index; FGF21, fibroblast growth factor-21; GDF15, growth differentiation factor-15; HSI, hepatic steatosis index; IQR, interquartile ranges; ISR, integrated stress response; MRI-PDFF, magnetic resonance imaging-based proton density fat fraction; MSI-S, metabolic stress index for steatosis; NLFS, NAFLD liver fat score; NAFLD, Nonalcoholic fatty liver disease; NASH, nonalcoholic steatohepatitis; ROS, reactive oxygen species; TG, triglycerides; TC, total cholesterol; WHR, waist-to-hip ratio.
