# Supplementary material for: Metabolic Stress Index Including Mitochondrial Biomarker for Noninvasive Diagnosis of Hepatic Steatosis
Source: Front Endocrinol (Lausanne). 2022 May 19;13:896334. doi: 10.3389/fendo.2022.896334 (PMC9160793; doi:10.3389/fendo.2022.896334)
Supplement: Supplementary file 1 [file DataSheet_1.pdf]

Supplementary Material

1 Supplementary Methods

1.1 Study Participants

Development cohort

The study subjects were recruited from 1894 individuals who completed the 3rd follow-up survey (from May 2011 to October 2017) among those already enrolled in the population-based cohort study, KoGES-ARIRANG (the Korean Genome and Epidemiology Study on Atherosclerosis Risk of Rural Areas in the Korean General Population). Subjects with a clinical history of secondary causes of steatosis or alternative diagnoses were excluded, including old age (≥85 years old), alcohol abuse (men, >30 g/day; women, >20 g/day), malignancy, chronic viral hepatitis (hepatitis B and/or C), drug-induced liver injury, autoimmune liver disease and Wilson's disease. A total of 348 subjects were finally enrolled on a consecutive basis between November 2018 and February 2020, according to the order in which they responded to email or telephonic recruitment used in the present study. Among the 348 enrolled individuals, 343 participants (124 males and 219 females) were included in this analysis and 5 subjects were excluded (incomplete data). Based on comprehensive liver assessments, 135 patients with NAFLD and 199 control subjects were selected for the steatosis prediction model; 9 subjects with liver fibrosis alone were excluded.

Validation cohort

The subjects were drawn from 49 patients who had been subjected to liver biopsy and were admitted to Wonju College of Medicine University Hospital between September 2018 and July 2020 for the evaluation and management of NAFLD. In addition to the exclusion criteria applied in the development cohort, except for age (≥75 years old), those who did not provide informed consent; patients with severe liver failure (or hepatic encephalopathy), spontaneous bacterial peritonitis, hepatorenal syndrome, acute renal impairment, underlying severe cardiac illness, or non-cirrhotic portal hypertension; and patients using nonselective ß-blockers, nitrates, or any other pharmacotherapy for prevention of variceal bleeding were excluded from the study. Patients with fragmented and nodular-shaped biopsy specimens (<2 mm length, more than 3 pieces, or specimens which were too small for the interpretation of histological grade [<10 mm total length of specimen]) or patients with insufficient serum for biomarker testing were also excluded. Therefore, a total of 41 patients were included in the validation group.

Magnetic resonance imaging (MRI)-proton density fat fraction (PDFF)

MRI was performed with a 3T system (MAGNETOM Skyra; Siemens, Erlangen, Germany) equipped with a combination of 30-channel body and 32-channel spine matrix coils. Subjects were examined in a supine position, and all images were obtained during expiratory breath-hold. Multi-echo Dixon mapping was performed to evaluate the liver fat and iron content with the following parameters: echo times (TE) = 1.05 ms, 2.46 ms, 3.69 ms, 4.92 ms, 6.15 ms, and 7.38 ms; repetition time (TR) = 9.00 ms; flip angle = 4°; field of view (FOV) = 450 mm^2 × 393 mm^2; matrix size = 160 mm × 111 mm; slice thickness = 3.5 mm; number of slices = 72; controlled aliasing in parallel imaging results in higher acceleration factor =

2 × 2; acquisition time = 13 s. Screening Dixon and multi-echo Dixon sequences were performed sequentially. Water images, fat images, goodness of fit images, MRI-PDFF maps and reports of screening and multi-echo Dixon were acquired automatically. After two measurements were performed at a 2-week interval, one clinician recorded the fat fraction from multi-echo Dixon acquisition for each patient.

Histological evaluation

Ultrasound-guided percutaneous liver biopsy was performed using a needle biopsy gun (Acecut, TSK Laboratory, Japan) with a 16 gauge 11.5 cm needle and a 15 mm biopsy specimen notch. Biopsy tissues (5 micrometre sections; hematoxylin-eosin and Masson-trichrome staining), were interpreted by two hepato-pathologists who were blinded to the subjects' clinical and laboratory data. Along with the degree of steatosis, the NAFLD activity score (NAS) was calculated for each specimen to classify NAFLD into simple steatosis (NAS 0–2) or borderline steatosis/steatohepatitis (NAS 3–4) and steatohepatitis (NASH) (NAS ≥5). The NAS comprised of three histological features of NAFLD: steatosis (0 to 3), lobular inflammation (0 to 3), and hepatocyte ballooning (0 to 2).

Ultrasonography

All ultrasonography investigations were performed by one hepatologist using an Aplio i500 ultrasound machine (Canon Medical Systems, Otawara, Japan) with a low frequency convex transducer. The four ultrasonographic signs (abnormal hepatorenal echoes, loss of echogenicity of the portal vein, poor diaphragm visualization and posterior beam attenuation) were evaluated to determine the severity of fatty liver disease (1, 2).

Clinical and laboratory assessments

Prior to MR-based evaluation, anthropometric measurements and blood samples were obtained on the same day. Height and weight were measured with participants wearing lightweight clothing and no shoes. Waist circumference was measured midway between the lowest rib and the superior border of the iliac crest. Hip circumference was measured around the widest portion of the buttocks. The circumferences are given as the mean of two measurements to the nearest 0.1 cm. Body mass index (BMI) was calculated as the weight in kilograms divided by the square of the height in meters.

After fasting for at least 10 hours, blood samples were drawn from each participant from the antecubital vein for analyses of biochemical parameters and NAFLD-related biomarkers. The separated sera were immediately stored at -80 °C for further analysis. A calibrated Roche Cobas® 8000 modular analyser consisting of c702a and e801 modules was used to measure serum concentrations of biochemical analytes with the manufacturer's reagents and calibrators (Roche, Mannheim, Germany). Triglycerides (TG), total cholesterol (TC), high-density lipoprotein cholesterol (HDL-C), γ-glutamyltransferase (γ-GT), and uric acid were measured by enzymatic-colorimetric methods. Aspartate aminotransferase (AST), alanine aminotransferase (ALT), alkaline phosphatase (ALP), creatinine, albumin, blood urea nitrogen (BUN), protein, and total bilirubin were measured by colorimetric methods. Fasting blood glucose was measured by the hexokinase method. Insulin and C-peptide were determined by electro-chemiluminescence immunoassay. Phosphorus and calcium were measured by the Molybdate UV and

NM-BAPTA methods, respectively. Platelet count was analysed using an automated blood cell counter (ADVIA 2110I, Bayer, NY, USA).

The serum concentrations of FGF21, FGF19, growth differentiation factor (GDF) 15, adiponectin, leptin, retinol binding protein (RBP) 4, IL6, TGF-β1, and myostatin were quantified using the human Quantikine ELISA kits (R&D Systems, Minneapolis, MN, USA) and those of decorin were measured using the Raybio human DCN ELISA kit (RayBiotech, Norcross, GA, USA) according to the manufacturer's instructions. All the samples were analysed using assay kits from a single lot before the expiration date, and each assay was performed in four separate batches. All mean intra-assay and inter-assay coefficients of variation were <10 %.

The homeostatic model assessment index (HOMA-IR) was calculated as [fasting insulin (μU/mL) × fasting glucose (mg/dL)]/405. Diabetes was defined as a fasting blood glucose level ≥126 mg/dL, previous diagnosis of diabetes, or use of anti-diabetic drugs. Hypertension was defined as blood pressure ≥140/90 mmHg, previous diagnosis of hypertension, and/or use of anti-hypertensive medication. Dyslipidaemia was defined as total-cholesterol ≥240 mg/dL, HDL-cholesterol <40 mg/dL in men and <50 mg/dL in women, and/or use of an anti-dyslipidaemic treatment. Metabolic syndrome was defined according to the International Diabetes Federation criteria (3)— central obesity (waist circumference ≥90 cm in men and ≥80 cm in women) and the presence of at least 2 of the following components: (1) serum triglycerides ≥150 mg/dL or specific treatment for hypertriglyceridemia; (2) serum high-density lipoprotein (HDL) <40 mg/dL in men and <50 mg/dL in women or specific treatment for hypo-HDL-cholesterolemia; (3) systolic blood pressure (BP) ≥130 mmHg or diastolic BP ≥85 mmHg, or treatment for previously diagnosed hypertension; and (4) fasting plasma glucose >100 mg/dL or previously diagnosed type 2 diabetes.

Existing prediction indices of liver steatosis

The following non-invasive liver steatosis indices were calculated: the fatty liver index (FLI) (4), NAFLD liver fat score (NLFS) (5), and hepatic steatosis index (HSI) (6). All prediction indices for liver steatosis are freely available, as they use clinical and laboratory parameters, and have fair to good accuracy in their derivation populations. Although the diagnostic cut-off points were reported for the derivation population, in order to aim for maximum performance based on this cohort, we recalculated the optimum cut-off values of the scores. The respective equations are shown below.

Equations of the non-invasive prediction scores for liver steatosis

Indices	Equations
FLI	$= e^x / (1 + e^x) \times 100$, $x = 0.953\times \text{Log}_e(\text{TG}) + 0.139\times \text{BMI} + 0.718\times \text{Log}_e(\gamma\text{-GT}) + 0.053\times \text{WC} - 15.745$
NLFS	= -2.89 + 1.18×metabolic syndrome (yes =1, no = 0) + 0.45×diabetes mellitus (yes = 2, no = 0) + 0.15×insulin + 0.04×AST - 0.94×AST/ALT ratio
HSI	$= e^x / (1 + e^x) \times 100$, x = 0.315×BMI + 2.421×ALT/AST ratio + 0.630×diabetes mellitus (yes = 1, no = 0) - 9.960

FLI, fatty liver index; NLFS, NAFLD liver fat score; HSI, hepatic steatosis index; AST, aspartate-aminotransferase; ALT, alanine-aminotransferase; TG, triglyceride; γ-GT, γ-glutamyltransferase.

Statistical analysis

A univariate descriptive statistic was used to compare subjects with and without liver steatosis. Continuous data were tested for normality using Shapiro-Wilk tests and presented as median with interquartile range (IQR). Categorical data were presented as frequencies with proportion. Comparisons of continuous data between subgroups were conducted by the Student's *t*-test, Mann-Whitney's *U* test, or Kruskal Wallis test followed by Dunnett's T3 post hoc test, as appropriate. Categorical data were analysed using the chi-square test. Correlations between the continuous data were assessed by Spearman's correlation coefficient (r).

All variables were included in a multivariate forward stepwise logistic regression analysis to identify variables independently associated with the presence or absence of NAFLD. Those variables with P <0.05 by multivariate logistic regression analysis were used to construct predictive scoring systems. Non-parametric data were used as independent variables after natural logarithmic transformation. Overall model calibration was assessed using the Hosmer-Lemeshow goodness-of-fit test and global performance and predictability were tested using the Nagelkerke R^2. The contribution strength of each variable to the multivariate model was evaluated by the Wald chi-square value (Wald), which was calculated by squaring the ratio of the regression coefficient divided by its standard error.

Area under the receiver operator characteristic (AUROC) curves were calculated to evaluate the diagnostic performance of the prediction models. Other assessments like sensitivity, specificity, positive likelihood ratio (LR+), negative likelihood ratio (LR-), positive predictive value (PPV), and negative predictive value (NPV) were also used to estimate model performance. The diagnostic accuracy of the scoring system was assessed as follows:

Accuracy = sensitivity × prevalence + specificity × (1 - prevalence)

The AUROCs were compared using DeLong's method (7). The optimal cut-off values were determined by maximizing the sum of the sensitivity and specificity on the Youden index (8). To build the estimating equations of liver fat fraction, multivariate forward stepwise linear regression analysis was performed using the same variables employed in the scoring system for NAFLD. The ICC was calculated to assess the reliability of quantitative prediction. ICC values of 0.90–1.00, 0.75–0.90, 0.5–0.75, and 0–0.50 were considered excellent, good, moderate, and poor agreement, respectively (9). Bland-Altman plot was constructed to evaluate the agreement between the measured and estimated value (10). Data were analysed using SPSS 25.0 software (IBM Corp., Armonk, N.Y., USA). A 2-sided P value of less than 0.05 was considered statistically significant. All graphs were generated using GraphPad Prism 8.0 software (GraphPad Software, Inc., San Diego, CA, USA).

2 Supplementary Results

Prediction of Liver Fat Content

The equation for estimating liver fat content was deduced from the multivariate stepwise linear regression analysis using the same variables employed for the MSI-S:

Liver fat content (ln %) = 2.631·WHR + 0.18·ln(FGF21, pg/mL) – 0.131·ln(FGF19, pg/mL) – 0.125·ln(A/L, 10^3) + 0.102·ln(insulin, mU/L) + 2.039·ln(albumin, g/dL) + 0.288·ln(TG, mg/dL) + 0.344·ln(TC, mg/dL) + 0.27·ln(ALT, IU/L) – 8.426

The estimated liver fat content has good correlation and agreement with the measured PDFF using MRI. The adjusted R^2 of the model was 0.475 and the ICC was 0.79 (95% CI 0.74–0.83) (Supplementary Table S3 and Fig. S2A). A Bland-Altman plot showed that 94.9 % of the subjects had values that were within the limits of the mean difference (Supplementary Fig. S2B).

Supplementary Table S1. Clinical characteristics and biochemical values of subjects with and without hepatic steatosis.

Variable	No hepatic steatosis (MRI-PDFF <6.4)	Hepatic steatosis (MRI-PDFF ≥6.4)	P-value
n (M/F)	199 (63/136)	135 (56/79)	0.066
Liver fat (%, MRI-PDFF)	2.9 (2.2, 4.4)	10.4 (8.3, 14)	<0.001
Liver fat (ln %, MRI-PDFF)	1.1 (0.8, 1.5)	2.3 (2.1, 2.6)	<0.001
Age (years)	66 (61, 72)	65 (61, 71)	0.426
BMI (kg/m^2)*	24.1 (22.3, 25.8)	26.8 (24.9, 28.4)	<0.001
Waist (cm)	81 (76, 88)	89 (84, 95)	<0.001
WHtR*	0.52 (0.49, 0.55)	0.55 (0.53, 0.59)	<0.001
WHR*	0.88 (0.85, 0.92)	0.93 (0.9, 0.96)	<0.001
Systolic blood pressure (mmHg)	126 (112, 140)	129 (120, 140)	0.241
Diastolic blood pressure (mmHg)	80 (70, 84)	80 (76, 86)	0.127
Triglyceride (mg/dL)	112 (86, 165)	165 (123, 242)	<0.001
Total cholesterol (mg/dL)	173 (151, 198)	185 (154, 209)	0.031
High-density lipoprotein (mg/dL)	54 (44, 65)	48 (40, 58)	0.001
Fasting glucose (mg/dL)	99 (93, 105)	102 (95, 113)	0.012
Insulin (mU/L)	6 (4, 10)	11 (7, 21)	<0.001
HOMA-IR	1.5 (0.9, 2.6)	2.8 (1.7, 5.3)	<0.001
AST (IU/L)	22 (19, 25)	23 (20, 29)	0.040
ALT (IU/L)	17 (13, 21)	21 (17, 28)	<0.001
ALT/AST	0.77 (0.64, 0.92)	0.94 (0.77, 1.12)	<0.001
GGT (IU/L)	18 (13, 25)	25 (17, 46)	<0.001
ALP (IU/L)	69 (58, 82)	67 (55, 83)	0.869
Albumin (g/dL)	4.6 (4.5, 4.8)	4.6 (4.6, 4.8)	0.071
Uric acid (mg/dL)	4.5 (3.9, 5.3)	5.1 (4.1, 5.8)	<0.001
Total bilirubin (mg/dL)	0.4 (0.31, 0.55)	0.43 (0.32, 0.56)	0.375
Protein (g/dL)	7.4 (7.1, 7.6)	7.5 (7.3, 7.7)	0.002
Blood urea nitrogen (mg/dL)	16 (13, 19)	15 (13, 18)	0.210
Creatinine (mg/dL)	0.72 (0.63, 0.86)	0.77 (0.67, 0.93)	0.029
Phosphorus (mg/dL)	3.7 (3.4, 4)	3.8 (3.5, 4.1)	0.063
Calcium (mg/dL)	9.5 (9.3, 9.8)	9.7 (9.4, 9.9)	<0.001
C-Peptide (ng/mL)	1.9 (1.4, 2.7)	3 (2.1, 4.4)	<0.001
Growth differentiation factor 15 (pg/mL)	829.3 (672, 1142.8)	1015.9 (708.5, 1387.7)	0.002
Fibroblast growth factor 21 (pg/mL)	171.4 (112.3, 275.8)	306.8 (192.4, 486.4)	<0.001
Fibroblast growth factor 19 (pg/mL)	197.7 (119.4, 325.5)	148.4 (86.2, 248)	<0.001
Adiponectin (μg/mL)	7.08 (4.43, 12.6)	4.66 (2.57, 7.82)	<0.001
Leptin (ng/mL)	6.93 (3.65, 11.68)	10.13 (6.18, 16.35)	<0.001
Adiponectin-to-leptin ratio (10^3)	1.09 (0.55, 2.38)	0.46 (0.26, 0.76)	<0.001
Retinol binding protein 4 (μg/mL)	29.9 (25.1, 34.7)	31.6 (27.2, 38.9)	0.008
Interleukin 6 (pg/mL)	1.44 (1.04, 2.26)	1.91 (1.26, 2.72)	<0.001
Transforming growth factor-β1 (ng/mL)	23.6 (19.1, 29.3)	25.4 (20.5, 30.8)	0.025
Myostatin/GDF8 (ng/mL)	2.6 (1.97, 3.19)	2.58 (2, 3.17)	0.841

Decorin (ng/mL)	6.62 (5.45, 7.69)	6.33 (4.99, 7.72)	0.452
Fatty liver index	19.2 (9.8, 35.1)	49.7 (31.5, 68)	<0.001
NAFLD Liver Fat Score	-1.8 (-2.6, -0.57)	-0.1 (-1.35, 1.4)	<0.001
Hepatic steatosis index	0.41 (0.24, 0.61)	0.7 (0.5, 0.84)	<0.001
Central obesity (%)	86 (43)	102 (76)	<0.001
Dyslipidemia (%)	79 (40)	56 (42)	0.745
Hypertension (%)	89 (45)	73 (54)	0.093
Type 2 diabetes (%)	35 (18)	37 (27)	0.032
Metabolic syndrome (%)	67 (34)	88 (57)	<0.001
Drugs			
Antidiabetic agents	34 (17)	37 (27)	0.024
Lipid-lowering agents	68 (34)	46 (34)	0.985
Antihypertensive agents	88 (44)	73 (54)	0.077

MRI-PDFF, magnetic resonance imaging–proton density fat fraction; BMI, body mass index; WHtR, waist-to-height ratio; WHR, waist-to-hip ratio; HOMA-IR, homeostatic model assessment of insulin resistance; AST, aspartate-aminotransferase; ALT, alanine-aminotransferase; γ-GT, γ-glutamyltransferase; ALP, alkaline-phosphatase. Continuous variables are presented as median (IQR) and compared using the Mann-Whitney *U* test or independent *t*-test*. Categorical data are expressed as sample size (proportion) and analysed by Pearson's chi-squared test.

Supplementary Table S2. Clinico-biochemical parameters and histological characteristics of the biopsy-evaluated subjects in the validation cohort.

Variable	
n [M/F]	41 [16/25]
Age (years)	47.0 (32.5, 59)
BMI (kg/m^2)	27.7 (25.9, 31.3)
Waist circumference (cm)	93.8 (88, 102)
Hip circumference (cm)	101.0 (97.3, 107.8)
Waist-hip ratio	0.92 (0.88, 0.97)
Waist-height ratio	0.59 (0.53, 0.62)
Systolic blood pressure (mmHg)	130 (124, 133.5)
Diastolic blood pressure (mmHg)	78 (72.5, 83.5)
Triglyceride (mg/dL)	127 (94.5, 173)
Total cholesterol (mg/dL)	169 (141.5, 189.5)
High-density lipoprotein (mg/dL)	43 (35.5, 51)
Fasting glucose (mg/dL)	101 (93.5, 119.5)
Insulin (mU/L)	13.1 (8.5, 21.5)
HOMA-IR	3.62 (2.27, 5.65)
AST (IU/L)	38 (30, 58)
ALT (IU/L)	58 (32.5, 84)
AST/ALT	0.62 (0.53, 1.06)
ALT/AST	1.63 (0.94, 1.9)
γ-GT (IU/L)	52 (29, 73.5)
ALP (IU/L)	68 (53.5, 91.5)
Albumin (g/dL)	4.3 (4.1, 4.5)
Fibroblast growth factor 21 (pg/mL)	409.8 (267.1, 634.7)
Fibroblast growth factor 19 (pg/mL)	90.6 (39.76, 154.51)
Adiponectin (μg/mL)	2.85 (2.38, 3.77)
Leptin (ng/mL)	16.56 (9.5, 22.24)
A/L (10^3)	0.164 (0.108, 0.316)
Central obesity (n)	37 (90.2)
Dyslipidemia (n)	11 (26.8)
Hypertension (n)	12 (29.3)
Type 2 diabetes (n)	15 (36.6)
Metabolic syndrome (n)	28 (68.3)
Grade of steatosis: S0/S1/S2/S3 (n)	3/13/16/9 (7.3/31.7/39.0/22.0)
Lobular inflammation: I0/I1/I2/I3 (n)	13/18/9/1 (31.7/43.9/22.0/2.4)
Hepatocyte ballooning: B0/B1/B2 (n)	19/19/3 (46.3/46.3/7.4)
NAFLD activity score [median, (min-max)]	3 (0, 7)

HOMA-IR, homeostatic model assessment of insulin resistance; AST, aspartate-aminotransferase; ALT, alanine-aminotransferase; γ-GT, γ-glutamyltransferase; ALP, alkaline-phosphatase. Continuous variables and categorical data are presented as median (interquartile range) and sample size (proportion), respectively.

Supplementary Table S3. Multivariate linear regression model for estimation of liver fat content.

Variable	B (95% CI)	SE	β	*t*	P-value	VIF
WHR	2.631 (1.494–3.769)	0.578	0.201	4.551	<0.001	1.237
Ln [triglyceride (mg/dL)]	0.288 (0.152–0.424)	0.069	0.196	4.157	<0.001	1.411
Ln [total cholesterol (mg/dL)]	0.344 (0.021–0.667)	0.164	0.089	2.094	0.037	1.134
Ln [fasting insulin (mU/L)]	0.102 (0.003–0.202)	0.051	0.106	2.020	0.044	1.761
Ln [ALT (IU/L)]	0.27 (0.113–0.427)	0.080	0.147	3.383	<0.001	1.194
Ln [albumin (g/dL)]	2.039 (0.728–3.35)	0.666	0.128	3.059	0.002	1.108
Ln [FGF21 (pg/mL)]	0.18 (0.098–0.263)	0.042	0.187	4.289	<0.001	1.206
Ln [FGF19 (pg/mL)]	-0.131 (-0.212–-0.051)	0.041	-0.135	-3.206	0.001	1.124
Ln [A/L (10^3)]	-0.125 (-0.189–-0.061)	0.033	-0.191	-3.849	<0.001	1.563
Constant	-8.426 (-11.253–-5.6)	1.437		-5.865	<0.001	
Model summary	F(9, 324) = 34.494; p = 2.2E-42; Durbin-Watson score: 1.913<br>R^2 = 0.489; Adjusted R^2 = 0.475, SEE = 0.565					

B, unstandardized regression coefficient; SE, standard error; SEE, standard error of estimate; β, standardized regression coefficient; VIF, variance inflation factor; ln, natural logarithm; WHR, waist-to-hip ratio; ALT, alanine-aminotransferase; A/L, adiponectin-to-leptin ratio; FGF21, fibroblast growth factor 21; FGF19, fibroblast growth factor 19.
Dependent variable: liver fat content (ln %, MRI-PDFF)

Supplementary Table S4. Sensitivities and specificities of predictive indices identifying hepatic steatosis using thresholds with sensitivity and specificity of 90 %.

Development cohort	Thresholds	SN (%)	SP (%)	LR+	LR-	PPV (%)	NPV (%)	Accuracy (%)	TP and TN (%)	Indeterminate (%)	FP and FN (%)
**MSI-S**	23.9	90 (84–95)	67 (60–73)	2.7 (2.2–3.3)	0.14 (0.09–0.24)	65 (60–79)	91 (86–95)	76 (71–81)	65.9	24.5	9.6
	60.8	64 (56–73)	91 (86–94)	6.8 (4.3–10.5)	0.39 (0.31–0.50)	82 (75–88)	79 (75–83)	80 (75–84)			
**FLI**	18.5	90 (84–95)	47 (40–54)	1.7 (1.5–2.0)	0.20 (0.12–0.35)	54 (50–57)	88 (81–93)	65 (59–70)	44.6	45.8	9.6
	54.5	41(32–50)	91 (86–94)	4.3(2.7–6.9)	0.66 (0.57–0.76)	74 (64–82)	69 (66–72)	70 (65–75)			
**NLFS**	-2.03	90 (84–95)	41 (34–48)	1.5 (1.4–1.8)	0.23 (0.14–0.40)	51 (48–54)	86 (79–92)	61 (56–66)	39.2	51.2	9.6
	0.52	36 (28–45)	91 (86–94)	3.8 (2.4–6.2)	0.70 (0.62–0.81)	72 (61–81)	68 (65–71)	69 (63–74)			
**HSI**	37.9	90 (84–95)	41 (34–48)	1.5 (1.4–1.8)	0.23 (0.14–0.40)	51 (48–54)	86 (79–92)	61 (56–66)	37.7	52.7	9.6
	79.4	33 (25–41)	91 (86–94)	3.4 (2.1–5.6)	0.75 (0.66–0.85)	70 (59–79)	66 (64–69)	67 (62–72)			
**Validation cohort**	**Thresholds**	**SN (%)**	**SP (%)**	**LR+**	**LR-**	**PPV (%)**	**NPV (%)**	**Accuracy (%)**	**TP and TN (%)**	**Indeterminate (%)**	**FP and FN (%)**
**MSI-S**	23.9	100 (86–100)	19 (4–46)	1.2 (1.0–1.6)	-	64 (58–69)	100	66 (50–90)	65.9	14.6	19.5
	60.8	96 (80–99)	50 (25–75)	1.9 (1.2–3.2)	0.08 (0.01–0.58)	73 (62–82)	90 (55–99)	77 (61–89)			
**FLI**	18.5	84 (64–96)	31 (11–59)	1.2 (0.8–1.8)	0.51 (0.16–1.63)	63 (54–72)	58 (30–82)	62 (46–77)	46.3	31.7	22.0
	54.5	68 (47–85)	44 (20–70)	1.2 (0.7–2.0)	0.73 (0.33–1.62)	63 (51–74)	49 (30–68)	58 (42–73)			
**NLFS**	-2.03	100 (86–100)	19 (4.1–46)	1.2 (1.0–1.6)	-	64 (58–69)	100	66 (50–90)	58.5	19.5	22.0
	0.52	84 (64–96)	44 (20–70)	1.5 (0.9–2.4)	0.37 (0.13–1.05)	68 (57–77)	66 (40–85)	67 (51–81)			
**HSI**	37.9	100 (86–100)	6 (0.2–30)	1.1 (0.9–1.2)	-	61 (57–63)	100	61 (45–76)	53.6	17.1	29.3
	79.4	84 (64–96)	25 (7.3–52)	1.1 (0.8–1.6)	0.64 (0.19–2.20)	61 (53–69)	53 (24–79)	60 (43–75)			

Data are presented as percentages (95% CI)

MSI-S, metabolic stress index for liver steatosis; FLI, fatty liver index; NLFS, NAFLD liver fat score; HSI, hepatic steatosis index; SN, sensitivity; SP, specificity; LR+, positive likelihood ratio; LR–, negative likelihood ratio; PPV, positive predictive value; NPV, negative predictive value; TP, true positive; TN, true negative; FP, false positive; FN, false negative.

Supplementary Table S5. Correlation matrix of independent variables in MSI-S with all collected parameters.

Variable		MRI-PDFF	WHR	TG	TC	Insulin	ALT	Albumin	FGF21	FGF19	A/L
MRI-PDFF (%)	*r*	1.0	**0.409**	**0.438**	**0.127**	**0.424**	**0.392**	**0.146**	**0.439**	**-0.214**	**-0.462**
	p	-	**<0.001**	**<0.001**	**0.020**	**<0.001**	**<0.001**	**0.008**	**<0.001**	**<0.001**	**<0.001**
WHR	*r*		1.0	**0.206**	-0.101	**0.371**	**0.209**	-0.071	**0.318**	-0.081	**-0.185**
	p		-	**<0.001**	0.066	**<0.001**	**<0.001**	0.193	**<0.001**	0.139	**0.001**
TG (mg/dL)	*r*			1.0	**0.141**	**0.438**	**0.256**	-0.015	**0.303**	0.095	**-0.344**
	p			-	**0.010**	**<0.001**	**<0.001**	0.781	**<0.001**	0.082	**<0.001**
TC (mg/dL)	*r*				1.0	**-0.133**	0.057	0.094	**0.109**	**0.119**	**-0.129**
	p				-	**0.015**	0.300	0.087	**0.047**	**0.030**	**0.019**
Insulin (mU/L)	*r*					1.0	0.293	**-0.127**	**0.170**	-0.014	**-0.516**
	p					-	<0.001	**0.020**	**0.002**	0.805	**<0.001**
ALT (IU/L)	*r*						1.0	**0.137**	**0.117**	-0.084	**-0.302**
	p						-	**0.012**	**0.032**	0.127	**<0.001**
Albumin (g/dL)	*r*							1.0	0.082	**-0.149**	-0.048
	p							-	0.136	**0.006**	0.382
FGF21 (pg/mL)	*r*								1.0	-0.015	**-0.182**
	p								-	0.779	**0.001**
FGF19 (pg/mL)	*r*									1.0	**0.122**
	p									-	**0.026**
A/L ($\times 10^3$)	*r*										1.0
	p										-
Age (years)	*r*	-0.089	**0.228**	-0.047	**-0.242**	**0.119**	-0.074	**-0.254**	0.064	**0.167**	**0.194**
	p	0.105	**<0.001**	0.389	**<0.001**	**0.030**	0.176	**<0.001**	0.244	**0.002**	**<0.001**
BMI (kg/m^2)	*r*	**0.464**	**0.503**	**0.185**	0.045	**0.440**	**0.271**	-0.019	**0.224**	**-0.178**	**-0.484**
	p	**<0.001**	**<0.001**	**0.001**	0.417	**<0.001**	**<0.001**	0.730	**<0.001**	**0.001**	**<0.001**
WC (cm)	*r*	**0.425**	**0.799**	**0.211**	-0.060	**0.437**	**0.295**	-0.085	**0.256**	**-0.127**	**-0.327**
	p	**<0.001**	**<0.001**	**<0.001**	0.274	**<0.001**	**<0.001**	0.121	**<0.001**	**0.020**	**<0.001**
WHtR	*r*	**0.375**	**0.734**	**0.127**	-0.037	**0.389**	**0.191**	-0.083	**0.301**	-0.094	**-0.354**
	p	**<0.001**	**<0.001**	**0.020**	0.497	**<0.001**	**<0.001**	0.131	**<0.001**	0.086	**<0.001**
SBP (mmHg)	*r*	0.041	**0.132**	**0.111**	-0.034	**0.190**	**0.121**	-0.057	**0.133**	0.073	-0.078
	p	0.456	**0.015**	**0.042**	0.536	**<0.001**	**0.027**	0.303	**0.015**	0.182	0.153
DBP (mmHg)	*r*	0.082	0.054	**0.114**	0.102	**0.115**	**0.155**	0.032	**0.110**	0.038	-0.080
	p	0.134	0.329	**0.038**	0.063	**0.036**	**0.005**	0.556	**0.044**	0.492	0.147
HDL-C (mg/dL)	*r*	**-0.263**	**-0.270**	**-0.494**	**0.295**	**-0.325**	**-0.142**	**0.109**	**-0.123**	-0.037	**0.187**
	p	**<0.001**	**<0.001**	**<0.001**	**<0.001**	**<0.001**	**0.009**	**0.046**	**0.025**	0.501	**0.001**
Glucose (mg/dL)	*r*	**0.148**	**0.254**	**0.223**	**-0.166**	**0.460**	**0.116**	-0.005	0.053	-0.007	**-0.172**
	p	**0.007**	**<0.001**	**<0.001**	**0.002**	**<0.001**	**0.034**	0.927	0.333	0.900	**0.002**
HOMA-IR	*r*	**0.415**	**0.379**	**0.441**	**-0.150**	**0.987**	**0.293**	**-0.117**	**0.170**	-0.024	**-0.503**
	p	**<0.001**	**<0.001**	**<0.001**	**0.006**	**<0.001**	**<0.001**	**0.032**	**0.002**	0.667	**<0.001**
ALT (IU/L)	*r*	-0.029	-0.039	0.008	0.079	-0.006	**0.150**	-0.065	-0.044	-0.001	-0.067
	p	0.595	0.481	0.886	0.152	0.910	**0.006**	0.234	0.423	0.992	0.225

AST (IU/L)	*r*	**0.120**	0.044	0.038	0.001	0.104	**0.656**	0.025	**0.112**	0.030	-0.077
	p	**0.029**	0.425	0.489	0.995	0.058	**<0.001**	0.645	**0.041**	0.590	0.163
ALT/AST	*r*	**0.396**	**0.225**	**0.294**	0.064	**0.319**	**0.717**	**0.148**	0.054	**-0.121**	**-0.320**
	p	**<0.001**	**<0.001**	**<0.001**	0.247	**<0.001**	**<0.001**	**0.007**	0.325	**0.026**	**<0.001**
γ-GT (IU/L)	*r*	**0.373**	**0.282**	**0.423**	0.011	**0.338**	**0.509**	0.053	**0.326**	0.013	**-0.245**
	p	**<0.001**	**<0.001**	**<0.001**	0.846	**<0.001**	**<0.001**	0.335	**<0.001**	0.810	**<0.001**
Uric acid (mg/dL)	*r*	**0.257**	**0.152**	**0.240**	0.051	**0.198**	**0.163**	-0.019	**0.122**	0.037	-0.101
	p	**<0.001**	**0.005**	**<0.001**	0.351	**<0.001**	**0.003**	0.728	**0.026**	0.503	0.064
Total bilirubin (mg/dL)	*r*	0.093	**0.135**	**-0.168**	-0.025	**-0.132**	**0.126**	**0.157**	0.097	**-0.133**	0.083
	p	0.090	**0.014**	**0.002**	0.649	**0.016**	**0.022**	**0.004**	0.076	**0.015**	0.129
Protein (g/dL)	*r*	**0.166**	0.045	0.021	**0.172**	0.023	0.089	**0.458**	**0.166**	**-0.177**	-0.094
	p	**0.002**	0.415	0.699	**0.002**	0.671	0.106	**<0.001**	**0.002**	**0.001**	0.085
BUN (mg/dL)	*r*	**-0.117**	0.100	-0.097	-0.035	**0.109**	-0.067	**-0.108**	**-0.185**	**0.120**	0.077
	p	**0.033**	0.067	0.075	0.520	**0.047**	0.223	**0.048**	**0.001**	**0.029**	0.160
Creatinine (mg/dL)	*r*	**0.142**	**0.306**	**0.150**	**-0.145**	**0.235**	**0.166**	-0.023	0.050	0.067	0.036
	p	**0.009**	**<0.001**	**0.006**	**0.008**	**<0.001**	**0.002**	0.672	0.360	0.222	0.515
Phosphorus (mg/dL)	*r*	0.072	-0.014	**0.142**	**0.136**	0.028	0.008	0.091	0.096	-0.014	**-0.131**
	p	0.188	0.797	**0.009**	**0.013**	0.615	0.891	0.097	0.079	0.805	**0.016**
Calcium (mg/dL)	*r*	**0.205**	0.068	**0.127**	**0.124**	**0.119**	**0.209**	**0.500**	**0.209**	-0.079	**-0.173**
	p	**<0.001**	0.214	**0.020**	**0.024**	**0.029**	**<0.001**	**<0.001**	**<0.001**	0.149	**0.002**
C-peptide (ng/mL)	*r*	**0.396**	**0.385**	**0.472**	**-0.171**	**0.909**	**0.228**	**-0.145**	**0.226**	0.062	**-0.414**
	p	**<0.001**	**<0.001**	**<0.001**	**0.002**	**<0.001**	**<0.001**	**0.008**	**<0.001**	0.260	**<0.001**
GDF15 (pg/mL)	*r*	**0.150**	**0.341**	0.101	**-0.226**	**0.265**	**0.125**	**-0.171**	**0.227**	0.106	0.059
	p	**0.006**	**<0.001**	0.066	**<0.001**	**<0.001**	**0.022**	**0.002**	**<0.001**	0.052	0.282
Adiponectin (μg/mL)	*r*	**-0.384**	**-0.199**	**-0.361**	0.027	**-0.325**	**-0.260**	**-0.122**	**-0.109**	0.107	**0.684**
	p	**<0.001**	**<0.001**	**<0.001**	0.624	**<0.001**	**<0.001**	**0.026**	**0.047**	0.050	**<0.001**
Leptin (ng/mL)	*r*	**0.284**	0.082	**0.149**	**0.195**	**0.412**	**0.162**	-0.051	**0.167**	-0.065	**-0.703**
	p	**<0.001**	0.134	**0.006**	**<0.001**	**<0.001**	**0.003**	0.350	**0.002**	0.238	**<0.001**
RBP4 (μg/mL)	*r*	**0.187**	**0.108**	**0.165**	0.066	0.063	**0.163**	**0.255**	**0.248**	0.092	0.018
	p	**0.001**	**0.049**	**0.003**	0.230	0.251	**0.003**	**<0.001**	**<0.001**	0.092	0.741
IL6 (pg/mL)	*r*	**0.220**	**0.214**	0.076	-0.044	**0.133**	-0.021	**-0.221**	**0.300**	0.077	-0.051
	p	**<0.001**	**<0.001**	0.167	0.423	**0.015**	0.702	**<0.001**	**<0.001**	0.160	0.355
TGF-β1 (ng/mL)	*r*	**0.111**	0.098	0.079	**0.147**	0.097	0.063	0.049	0.048	-0.066	-0.084
	p	**0.043**	0.073	0.151	**0.007**	0.078	0.251	0.374	0.383	0.232	0.127
Myostatin (ng/mL)	*r*	-0.036	0.020	-0.055	-0.093	0.030	0.069	**-0.176**	-0.091	0.030	**0.154**
	p	0.514	0.719	0.319	0.091	0.586	0.211	**0.001**	0.096	0.583	**0.005**
Decorin (ng/mL)	*r*	-0.041	**-0.114**	**-0.116**	0.060	-0.100	0.034	0.053	**-0.135**	0.003	**0.150**
	p	0.450	**0.037**	**0.034**	0.272	0.068	0.533	0.333	**0.014**	0.961	**0.006**

Spearman's correlation coefficients (*r*) and corresponding *p*-values (*p*) are shown with significant correlations in bold. MRI-PDFF, magnetic resonance imaging–proton density fat fraction; BMI, body mass index; WC, waist circumference; WHtR, waist-to-height ratio; WHR, waist-to-hip ratio; SBP, systolic blood pressure; DBP, diastolic blood pressure; TG, triglyceride; TC, total cholesterol; HDL-C, high-density lipoprotein-cholesterol; HOMA-IR, homeostatic model assessment of insulin resistance; AST, aspartate-aminotransferase; ALT, alanine-aminotransferase; γ-GT, γ-glutamyltransferase; ALP, alkaline-phosphatase; A/L, adiponectin-to-leptin ratio; BUN, blood urea nitrogen; GDF15, growth differentiation factor 15; RBP4, retinol binding protein 4.

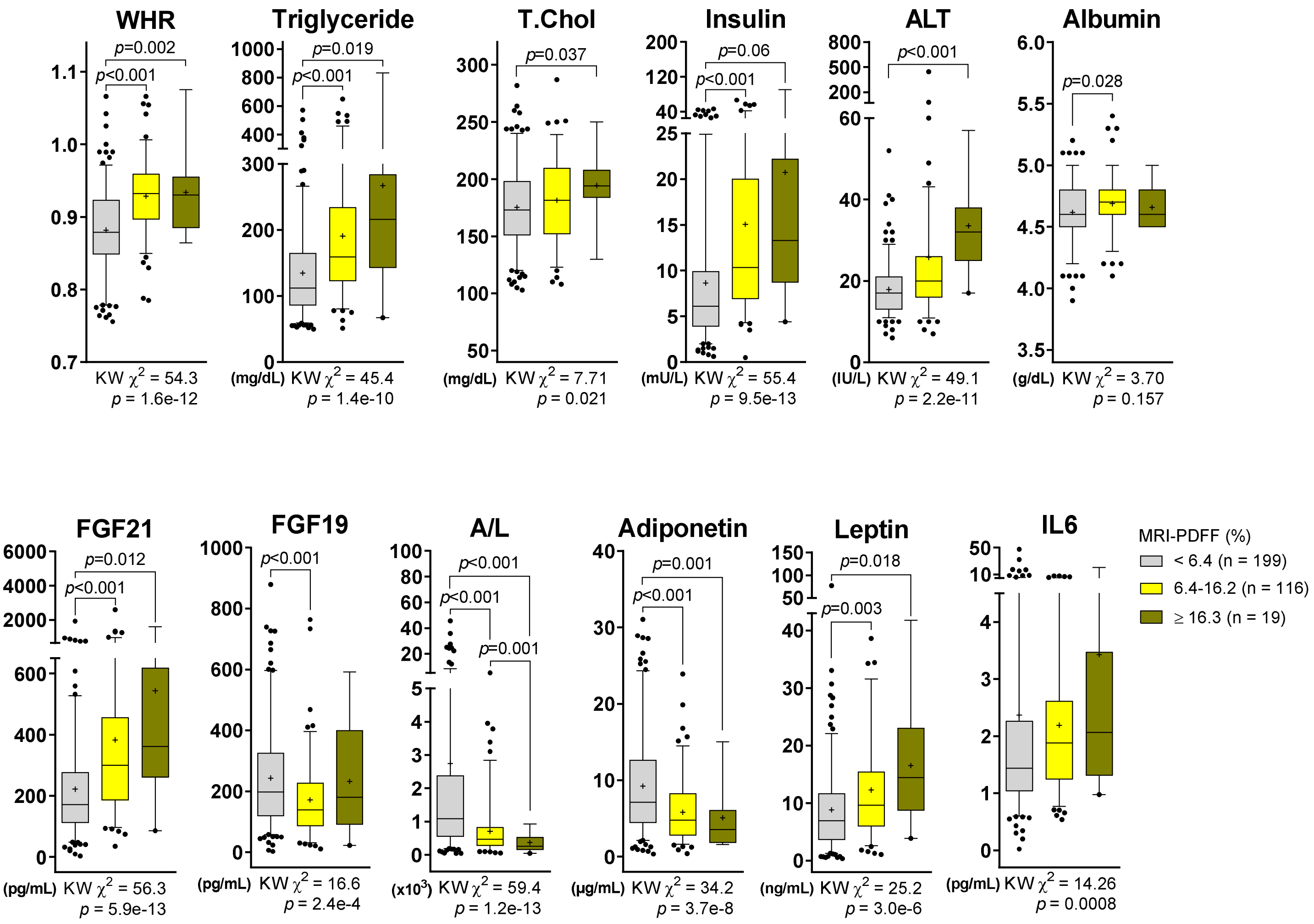

Supplementary Figure S1. Boxplot of steatosis predictors' values in relation to the fatty liver grades. Data are presented as box and whisker Tukey plots with medians and interquartile ranges (+, mean; •, outliers). Kruskal-Wallis (KW) test and post hoc Dunnett's T3 test revealed statistical differences. ALT, alanine-aminotransferase; A/L, adiponectin-to-leptin ratio; WHR, waist-to-hip ratio.

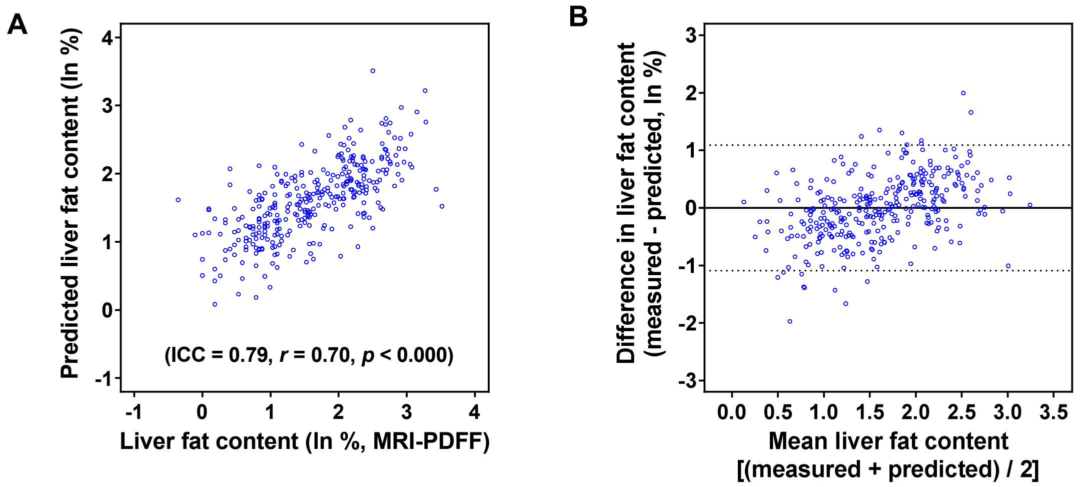

Supplementary Figure S2. Scatterplot (A) and Bland-Altman plot (B) show the relationships and agreement between MRI-PDFF values and predicted values by a multivariate linear regression model using biomarker variables. Mean difference and 95% limits of agreement (±1.96 S.D. of difference) are presented as black and dotted lines, respectively. MRI-PDFF, magnetic resonance imaging-based proton density fat fraction; ln, natural logarithm; ICC, intraclass correlation coefficient.

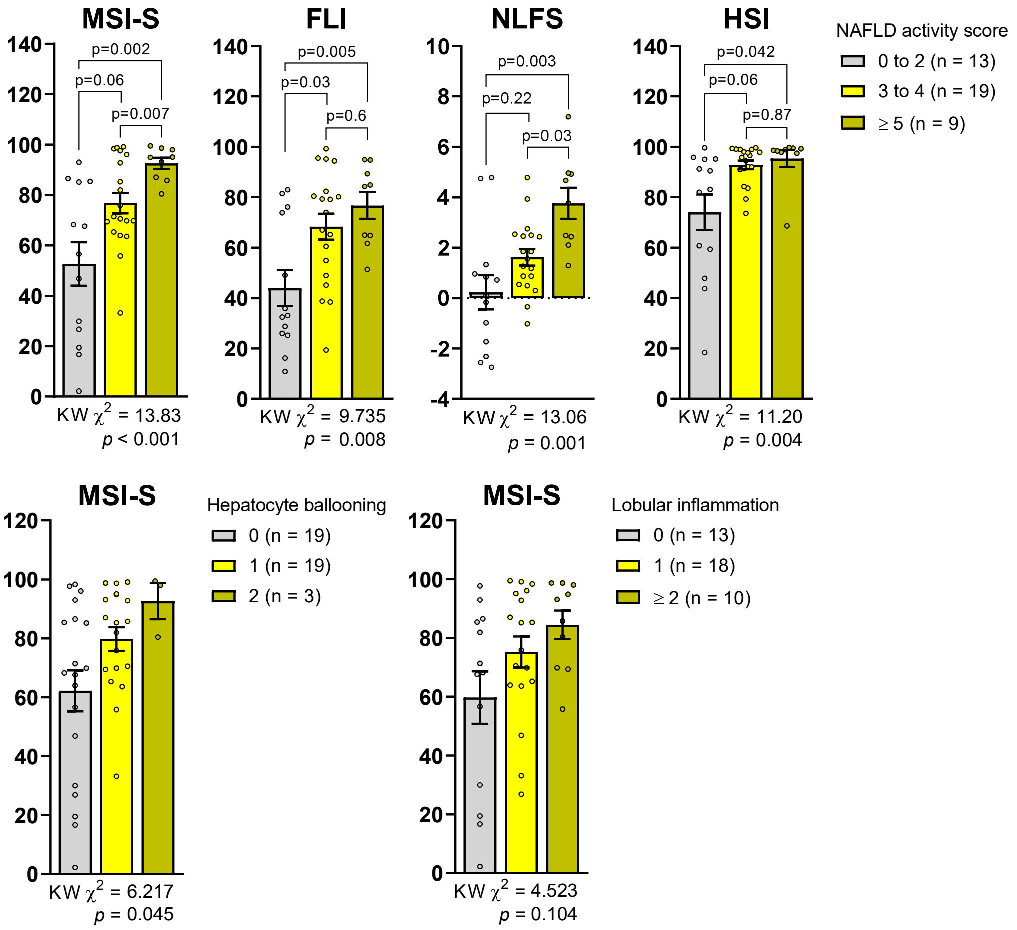

Supplementary Figure S3. Comparison of non-invasive prediction scores based on histological grades of NAFLD. Bars and circles represent the mean with standard error of the mean and individual values, respectively. Kruskal-Wallis (KW) test and post hoc Dunnett's T3 test revealed statistical differences. MSI-S, metabolic stress index of liver steatosis; FLI, fatty liver index; NLFS, NAFLD liver fat score; HSI, hepatic steatosis index.

Supplementary References

1. Mathiesen UL, Franzen LE, Aselius H, Resjo M, Jacobsson L, Foberg U, et al. Increased liver echogenicity at ultrasound examination reflects degree of steatosis but not of fibrosis in asymptomatic patients with mild/moderate abnormalities of liver transaminases. *Dig Liver Dis* (2002) 34:516-22. doi: 10.1016/s1590-8658(02)80111-6
2. Hamaguchi M, Kojima T, Itoh Y, Harano Y, Fujii K, Nakajima T, et al. The severity of ultrasonographic findings in nonalcoholic fatty liver disease reflects the metabolic syndrome and visceral fat accumulation. *Am J Gastroenterol* (2007) 102:2708-15. doi: 10.1111/j.1572-0241.2007.01526.x
3. Alberti KG, Zimmet P, Shaw J, Group IDFETFC. The metabolic syndrome--a new worldwide definition. *Lancet* (2005) 366:1059-62. doi: 10.1016/S0140-6736(05)67402-8
4. Bedogni G, Bellentani S, Miglioli L, Masutti F, Passalacqua M, Castiglione A, et al. The Fatty Liver Index: a simple and accurate predictor of hepatic steatosis in the general population. *BMC Gastroenterol* (2006) 6:33. doi: 10.1186/1471-230X-6-33
5. Kotronen A, Peltonen M, Hakkarainen A, Sevastianova K, Bergholm R, Johansson LM, et al. Prediction of non-alcoholic fatty liver disease and liver fat using metabolic and genetic factors. *Gastroenterology* (2009) 137:865-72. doi: 10.1053/j.gastro.2009.06.005
6. Lee JH, Kim D, Kim HJ, Lee CH, Yang JI, Kim W, et al. Hepatic steatosis index: a simple screening tool reflecting nonalcoholic fatty liver disease. *Dig Liver Dis* (2010) 42:503-8. doi: 10.1016/j.dld.2009.08.002
7. DeLong ER, DeLong DM, Clarke-Pearson DL. Comparing the areas under two or more correlated receiver operating characteristic curves: a nonparametric approach. *Biometrics* (1988) 44:837-45
8. Youden WJ. Index for rating diagnostic tests. *Cancer* (1950) 3:32-5. doi: 10.1002/1097-0142(1950)3:1<32::aid-cncr2820030106>3.0.co;2-3
9. Koo TK, Li MY. A Guideline of Selecting and Reporting Intraclass Correlation Coefficients for Reliability Research. *J Chiropr Med* (2016) 15:155-63. doi: 10.1016/j.jcm.2016.02.012
10. Bland JM, Altman DG. Statistical methods for assessing agreement between two methods of clinical measurement. *Lancet* (1986) 1:307-10
